# Pathway-specific inhibition of primaquine metabolism by chloroquine/quinine

**DOI:** 10.1186/s12936-016-1509-x

**Published:** 2016-09-13

**Authors:** Pius S. Fasinu, Babu L. Tekwani, Bharathi Avula, Narayan D. Chaurasiya, N. P. Dhammika Nanayakkara, Yan-Hong Wang, Ikhlas A. Khan, Larry A. Walker

**Affiliations:** 1 The National Center for Natural Products Research, School of Pharmacy, The University of Mississippi, University, MS 38677 USA; 2 Departments of BioMolecular Sciences, School of Pharmacy, The University of Mississippi, University, MS 38677 USA

**Keywords:** Chloroquine, Drug–drug interaction, Drug metabolism, Malaria, Pharmacokinetics, Primaquine, Quinine

## Abstract

**Background:**

There has been some evidence to suggest that the addition of chloroquine (CQ) or quinine (QN) to 8-aminoquinoline (8-AQ) treatment regimens may increase the therapeutic efficacy of the 8-AQ and simultaneously mitigate against its haemolytic toxicity. However, both CQ and QN are considered effective, although perhaps moderate inhibitors of CYP2D6, an enzyme now regarded as necessary for primaquine (PQ) pharmacologic activity. An understanding of the influence of CQ and QN on the metabolism of PQ may shed light on the potential mechanisms of the beneficial interaction.

**Methods:**

Differential metabolism of PQ enantiomers by recombinant human CYP2D6, monoamine oxidase A (MAO), and cryopreserved human hepatocytes in the presence/absence of CQ and QN.

**Results:**

Both CQ and QN significantly inhibited the activity of CYP2D6. PQ depletion by MAO and human hepatocytes was not affected significantly by the presence of CQ and QN. CYP2D6-mediated hydroxylation was largely suppressed by both CQ and QN. The formation of the primary deaminated metabolites, including carboxyprimaquine (CPQ) and cyclized side chain derivative from the aldehyde (*m/z* 241), was not sensitive to the presence of CQ and QN. However, the appearance of the glucuronides of CPQ and PQ alcohol were significantly suppressed. CQ and QN also inhibited the appearance of the *m/z* 257 metabolite with a similar pattern, suggesting that it may be derived from the CPQ conjugate. The apparent quinone-imine of CPQ (*m/z* 289) was only partially suppressed by both QN and CQ, but with a differential pattern of inhibition for the two drugs. The *m/z* 274 (quinone-imine of a ring-hydroxylated PQ metabolite) and *m/z* 422 (an apparent glucose conjugate of PQ) metabolites in hepatocytes were strongly suppressed by both QN and CQ, perhaps a reflection of the 2D6 inhibition by these drugs. The formation of the carbamoyl glucuronide of PQ (*m/z* 480) was not affected by CQ/QN.

**Conclusion:**

The metabolite-specific interactions in the current studies seem at variance with earlier reports of the dependence of PQ on CYP2D6 metabolism, and enhanced PQ anti-malarial activity/reduced toxicity in the presence of CQ/QN. These results suggest a complex picture in which CQ/QN may shift metabolite pathway balances towards a profile that retains efficacy, while reducing the formation or availability of toxic metabolites to erythrocytes. Alternatively, these drugs may alter transport or distribution of PQ metabolites in a fashion that reduces toxicity while maintaining efficacy against the parasite.

## Background

There has been an intense renewal of interest in primaquine (PQ) after over 60 years since its introduction into clinical medicine [1, 2]. This is understandably so because, despite spirited research efforts, no suitable alternative has yet been approved to rival the unique therapeutic profile of PQ. As the prototype 8-aminoquinoline (8-AQ), PQ is the only licensed drug capable of treating the relapsing liver stages (hypnozoites) of *Plasmodium vivax* [3]. It is gametocytocidal against *Plasmodium falciparum* and capable of blocking transmission. Additionally, PQ has been used as a prophylactic option against all forms of malaria [4]. It has found use in the treatment of artemisinin-resistant *P. falciparum* strains [5]. The major limitation in the clinical utility of PQ is its haemolytic toxicity in individuals with genetic deficiency in glucose-6-phosphate dehydrogenase (G6PD). Both the therapeutic and haemotoxic effects of PQ have been strongly linked to its metabolite(s) [6–8]. While a number of human metabolites of PQ have been described [9], the identity of the specific PQ metabolites responsible for efficacy and toxicity are not well understood [10].

Interactions between various anti-malarial drugs have been reported to improve efficacy and mitigate toxicity of PQ. In particular, there have been a number of clinical evidences presented over the years showing that quinine (QN) and chloroquine (CQ) can favourably affect the efficacy of 8-AQs; further, there are intriguing suggestions that the haemolytic toxicity of the 8-AQ class can be reduced. For instance, the addition of CQ increased the effectiveness of PQ and pamaquine while reducing the associated toxicity in Korean patients [11]. In a study to assess the toxicity of pentaquine, using doses two- to three-fold higher than therapeutic doses, ten patients were experimentally infected with malaria. Each patient received 120 mg pentaquine with or without QN [12]. Five subjects receiving pentaquine only experienced marked drops in haemoglobin (average 2.6 g/dl), but the five concomitantly receiving QN showed no change in haemoglobin. Moreover, no patient in the pentaquine/QN group had a relapse in 12 months compared to two in the pentaquine-only group, who had relapses in 12 and 26 days, respectively. Associated side effects of syncope and postural hypotension were not reported in the pentaquine/QN group compared to pentaquine-only group [12]. Thus, this study not only demonstrated the attenuation of the toxic effects of pentaquine by QN, it showed that QN improved the therapeutic efficacy of pentaquine. A similar observation, including an increase in plasma pentaquine concentration in the presence of QN, was reported by Alving and co-workers [13]. Being an 8-AQ like PQ, whose mechanism of toxicity might be similar, this observation suggests similar beneficial drug interactions with PQ. Since the pharmacological activity of the 8-AQs are dependent on their metabolites, the knowledge of the roles of each metabolites may be deductible from observed pharmacodynamic changes due to inhibition or facilitation of the formation of known metabolites by other agents.

The potential mechanisms underlying these interactions are unknown at present, but could be presumptively metabolism-linked. Until recently, the focus on PQ metabolites in clinical studies has been towards carboxy-primaquine (CPQ), which has been reported to be the major circulating metabolite [14]. In one study conducted to investigate interactions among anti-malarial drugs, QN caused a decrease in the area under the curve (AUC_0–24h_) of CPQ by about 50 % [15]. More recently, Pukrittayakamee et al. [16] conducted an open-label, cross-over, pharmacokinetic interaction study in 16 health human subjects. Volunteers received single doses of 30 mg PQ, 600 mg CQ, or the combination thereof. In contrast to the QN results, CQ caused a significant increase in the plasma levels of both PQ and its major metabolite, CPQ. Thus, these two drugs, while sharing a beneficial effect on PQ efficacy and toxicity, apparently do so by affecting mechanisms other than the monoamine oxidase-mediated metabolism of PQ to CPQ. Other pathways of PQ metabolism, now known to be prominent in human liver—but so far difficult to pinpoint their ultimate disposition—have not been explored from the perspective of CQ and QN effects.

More recent studies with PQ have developed compelling evidence that the metabolism to its active metabolites is dependent on the activity of cytochrome P450 2D6 (CYP2D6) [7, 17]. This evidence is based on the failure of PQ therapy in CYP2D6-null metabolizers and the ablation of the anti-malarial effect of PQ in CYP2D knockout mice. Thus, it would naturally be expected that inhibition of CYP2D6 would impair the efficacy of PQ. However, both QN and CQ have been shown to be moderate inhibitors of CYP2D6 with significant effects at clinically used doses [18, 19]. It is puzzling then, that they do not effectively antagonize the anti-malarial effects of PQ, and rather are potentiators. This suggests that either the putative dependence of PQ efficacy on CYP2D6 is incorrect, or that CQ and QN may be acting by other mechanisms.

Recent studies of PQ metabolism have revealed interesting new information about the metabolic pathways, suggesting that in humans the two enantiomers of PQ (which is used as a racemic mixture) are differentially metabolized to CPQ [20]. While the *R*(–) form of PQ is readily converted to CPQ, the *S*(+) form is much less so, and in fact >95 % of the CPQ circulating is attributable to *R*(–) PQ. This raises the question as to the primary metabolic route for the *S*(+) form. Furthermore, other studies revealed several other major metabolites of PQ generated in human hepatocytes [21] and some of these are formed predominantly or exclusively from one enantiomer of PQ [9, 21].

Therefore, the objective of the current study was to investigate the effects of CQ and QN on the clearance and metabolism profiles of PQ and its individual enantiomers by human CYP2D6, monoamine oxidase (MAO) and primary human hepatocytes.

## Methods

### Substrates, chemicals and co-factors

Racemic, (*S*)-(+)- and (*R*)-(−)- enantiomers of ^13^C(6)-labelled PQ (MW 259.1317 g/mol); PQ alcohol, 2-, 3-, and 4-hydroxyprimaquine and CPQ were synthesized as previously reported [9, 22–24]. The identity of the compounds synthesized was confirmed by spectral infra-red (IR), nuclear magnetic resonance (NMR), high-resolution mass spectrometry and physical data in comparison with published values.

Co-factors for enzyme activity, including glucose-6-phosphate and its dehydrogenase, reduced nicotinamide adenine dinucleotide phosphate and magnesium chloride were purchased from Sigma-Aldrich (St Louis, MO, USA). Analytical grade solvents, including acetonitrile and methanol were purchased from Fisher Scientific (Fair Lawn, NJ, USA). Milli-Q system (Millipore, Bedford, MA, USA) was used to purify water for analytical utility.

### Metabolic assay systems

Recombinant cytochrome-P450 supersomes containing CYP2D6 (1 nmol CYP/mL), and recombinant monoamine oxidase-A (MAO) were purchased from BD Biosciences (Billerica, MA, USA) and stored at −80 °C until used. Cryopreserved, metabolism-qualified hepatocytes (mixed-gender pooled) were obtained from Corning Life Sciences (Woburn, MA, USA) and stored in liquid nitrogen tank until use. Just before use, the metabolic systems were thawed according to supplier instructions. Typically, the vials containing the cryopreserved metabolic systems were dipped (without submerging) into water bath set at 37 °C for not more than 2 min in order to defrost the contents. The vials are then wiped with 70 % alcohol before the contents are poured into the recovery medium or incubation buffers.

### Incubations with CYP2D6 and monoamine oxidase

Typical assay with rCYP2D6 and MAO involved the dilution of the thawed enzymes with 0.1 mM phosphate buffered saline (pH 7.4), aliquot dispensing in clear 96-well plates, and the subsequent addition of the substrate (PQ) and the co-factor solution to initiate metabolic reactions. The final concentrations of additives in the incubation mixture of 500 µL are as follows: 20 µM PQ, 0.5 mg/mL of enzyme (CYP2D6 or MAO-A), 1.0 unit/ml of G6PD, 1.3 mM reduced nicotinamide adenine dinucleotide phosphate and 3.3 mM each of glucose-6-phosphate and magnesium chloride. Stock solutions of CQ/QN was prepared in water and added to the wells to a final concentration of 5 µM; an equivalent volume of buffer was included in control wells. Metabolic activity of the enzymes was halted at pre-determined times by the addition of one-part ice-cold methanol containing 0.5 µg/mL D_3_-primaquine as internal standard. The samples were kept on ice for an hour and then centrifuged (14,000 rpm; 4 °C; 10 min). Clear supernatants were collected for ultra-performance liquid chromatography-mass spectrometry (UHPLC-MS) analysis. All assays were performed in duplicate to observe intra-assay variations and repeated at separate days for inter-day comparisons. Controls included substrate incubation without enzyme or start solutions and incubations without the substrate.

### Hepatocyte incubations

Suspensions of pooled human hepatocytes were thawed from cryopreserved vials and recovered with the supplier-provided recovery media (containing buffered saline with 2 % fetal bovine serum and 300 mM glucose). This was followed by centrifugation (300*g*, 5 min) to pellet the cells, which were then re-suspended in the plating media provided by the suppliers. Cell viability was determined based on cell counts using a Bio-Rad automated cell counter (Hercules, CA, USA). The cell density was adjusted to approximately 1 million viable cells/mL in the plating media and incubated with 20 µM PQ at 37 °C in a humidified atmosphere of 95 % air and 5 % CO2 in an Eppendorf incubator (Hauppauge, NY, USA) with an attached shaker set at 100 rpm. In addition, incubation plates contained 5 µM CQ/QN or equal volume of the plating media. In parallel, substrate-free and cell-free incubations were performed as controls. At each time point, aliquots were taken for cell viability measurement. Reactions were quenched using two volumes of ice-cold methanol containing 0.5 µg/mL 6-D_3_-primaquine as internal standard at pre-determined time points (between 0 and 120 min).

Once metabolic reactions were quenched, the incubation mixtures were vortexed and kept at −20 °C for at least 4 h before further analysis. Clear supernatants of centrifuged samples (10,000*g*, 4 °C, 10 min) were dried in a Speedvac^®^ and reconstituted in methanol for UHPLC-MS analysis.

### Detection, identification and quantification of metabolites

Methods for simultaneous analysis of PQ and its metabolites using the UHPLC-MS were reported earlier [25]. This involved the use of a BEH Shield RP18 column (100 mm × 2.1 mm ID, 1.7 mm) equipped with an LC-18 guard column (Vanguard 2.1 × 5 mm, Waters Corp, Milford, MA, USA) on an ACQUITY UHPLC™ system (Waters) to which a conditioned auto-sampler (at 20 °C) was attached. Metabolites earlier identified, based on retention time, high resolution mass and MS–MS fragmentation pattern, were individually searched for and profiled against time in the presence and absence of CQ/QN.

## Results

**Fig. 1:**
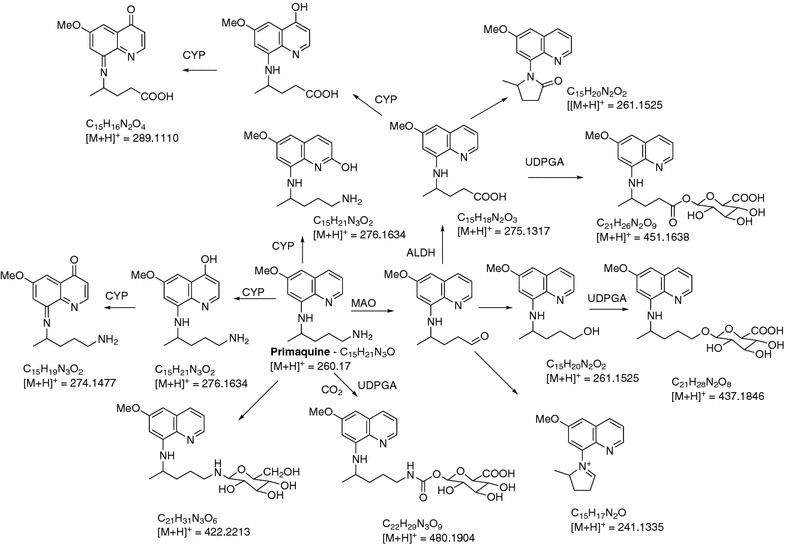
A putative pathway of PQ metabolism in human hepatocytes. Uridine-diphosphate-glucuronic acid which is co-factor necessary for glucuronidation catalyzed by UDPGT (Uridine-diphosphate glucuronosyltransferase); *CYP* cytochrome P450; *ALDH* aldehyde dehydrogenase) (First published by Fasinu et al. [21])

PQ is metabolized rapidly by both CYP2D6 and MAO. CYP2D6 is known to mediate ring oxidation and demethylation steps, while MAO catalyzes oxidative deamination of the side chain. The effect of 5 µM each of CQ and QN on the generation of known CYP2D6- and MAO-dependent metabolites of 20 µM PQ or its individual enantiomers was assessed using recombinant human enzymes. The influence of the two anti-malarial drugs on the generation of secondary metabolites in human hepatocytes was also investigated. In general, the oxidation of the quinoline ring proved very sensitive to the presence of CQ/QN, whereas the formation of the deaminated products was generally not markedly inhibited. The structures of the major metabolites are presented in Fig. 1.

### Influence of CQ and QN on CYP2D6-dependent PQ metabolites

**Fig. 2:**
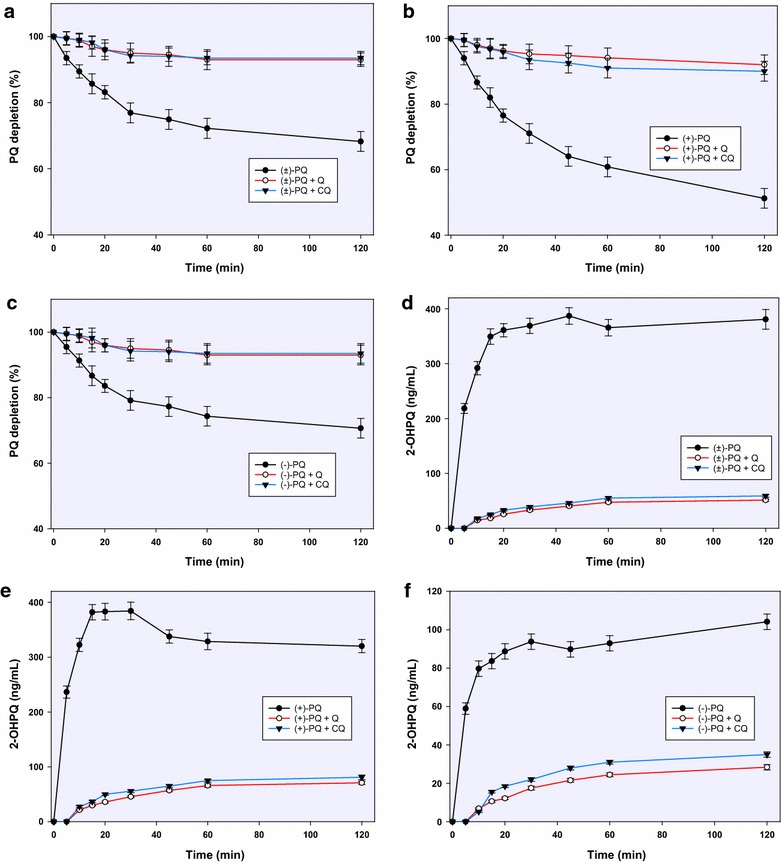
The influence of chloroquine and quinine on the CYP2D6-mediated depletion of racemic PQ (**a**) and its enantiomers (**b**, **c**); and formation of 2-hydroxyprimaquine from racemic PQ (**d**) and **e**, **f** over 2 h of incubation. Each *point* represents values: mean ± S.D. (n = 4)

PQ was steadily depleted to varying degrees in the incubation mixtures with recombinant CYP2D6 such that by 2 h, 65, 50 and 72 % of the racemic, (+)- and (−)-PQ remained, respectively. Both CQ and QN suppressed this depletion with all three substrates such that more than 90 % of the substrates remained after 2 h (Fig. 2a–c).

The major metabolites from CYP-2D6-mediated pathway were 2-, 3-, and 4-hydroxyprimaquine, 5,6-orthoquinone product from 5-hydroxyprimaquine and PQ alcohol (as earlier reported) [9]. The formation of 2-hydroxyprimaquine and the 5,6-orthoquinone predominated with (+)-PQ, while the 3-hydroxy- and 4-hydroxyprimaquine were formed more from the (−)-PQ. Quantitatively, 2-hydroxyprimaquine was four times more abundant from (+)-PQ than from (−)-PQ. Although the concentration of 2-hydroxyprimaquine peaked at 20 min with all substrates, 50 % of the peak concentration was already achieved by 5 min. CQ and QN completely suppressed the initial metabolite surge, with some metabolite appearing after an initial lag time (Fig. 2).

**Fig. 3:**
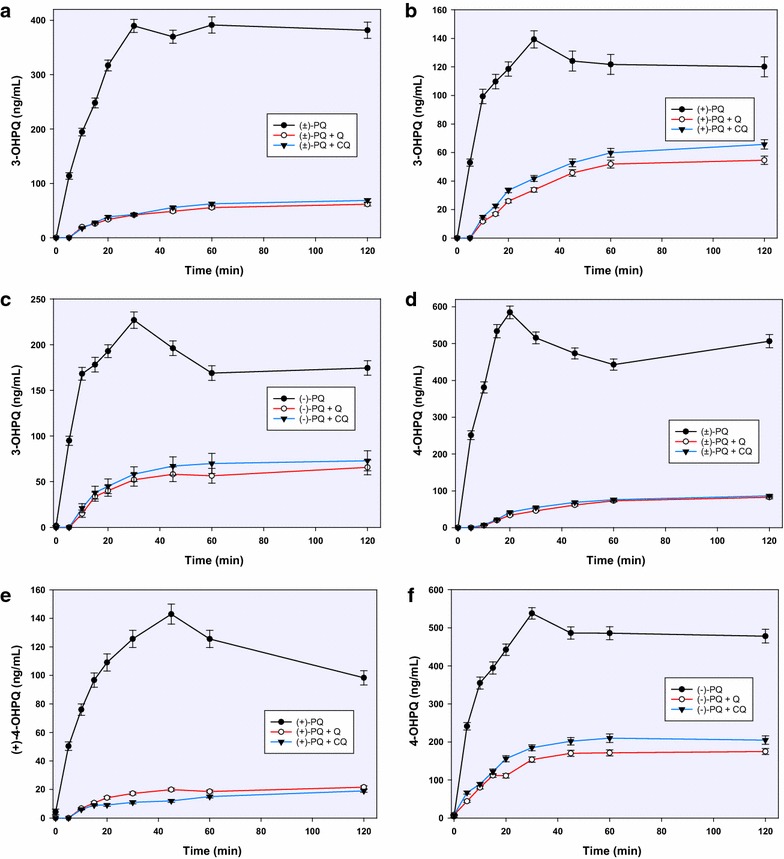
The influence of chloroquine and quinine on the CYP2D6-mediated formation and kinetics of 3-hydroxyprimaquine from racemic PQ (**a**) and its individual enantiomers (**b**, **c**); and on the formation of 4-hydroxyprimaquine from racemic PQ (**d**) and its enantiomers (**e**, **f**). Each *point* represents values mean ± S.D. (n = 4)

**Fig. 4:**
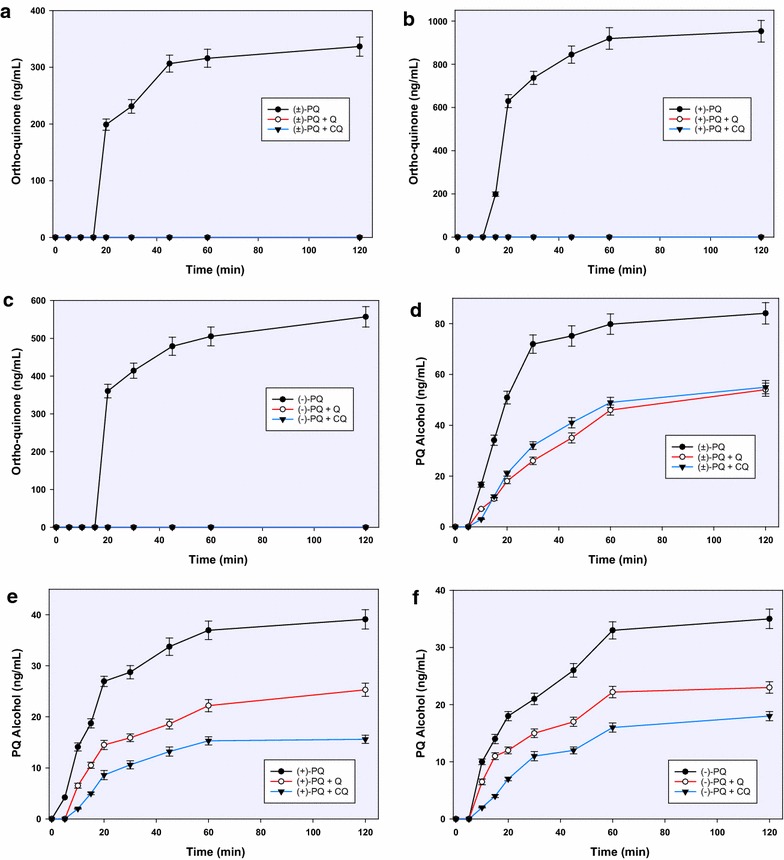
The inhibitory effect of chloroquine and quinine on the formation and time-course of the PQ 5,6-orthoquinone generated from racemic PQ (**a**) and its individual enantiomers (**b**, **c**) by CYP2D6; and on the formation of primaquine alcohol from CYP2D6-catalyzed metabolism of racemic PQ (**d**) and its enantiomers (**e**, **f**). Each point represents values mean ± S.D. (n = 4)

**Fig. 5:**
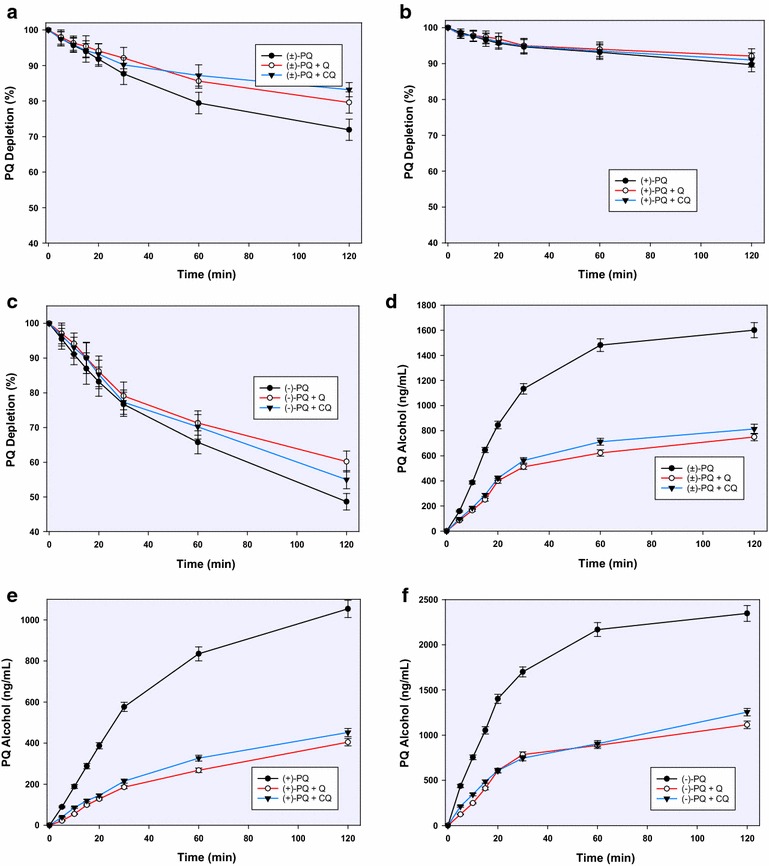
Comparative depletion of racemic PQ (**a**) and its enantiomers (**b**, **c**) on incubation with recombinant human monoamine oxidase; and the formation of PQ alcohol (**d**–**f**) from racemic PQ (**d**) and its enantiomers (**e**, **f**) by the same enzyme. Each *point* represents values mean ± S.D. (n = 4)

Unlike 2-hydroxyprimaquine, 3- and 4-hydroxyprimaquine were formed more readily from (−)-PQ than in (+)-PQ (2:1 and 5:1, respectively) (Fig. 3). Both 3-hydroxy- and 4-hydroxyprimaquine have sharp early rises, which were again completely suppressed by CQ and QN at the early stages, then appearing in limited amounts after 5 min. The inhibitory effect of QN on the formation of the monohydroxyprimaquines appeared to be slightly more than the equimolar CQ, but the two were generally comparable. Both CQ and QN completely suppressed the formation of the 5,6-orthoquinone (Fig. 4), presumptively derived from 5-hydroxyprimaquine. Both CQ and QN show only moderate inhibition on CYP2D6-catalyzed formation of the PQ terminal alcohol. Interestingly, in contrast to the effect on ring-hydroxylated metabolites, CQ caused greater inhibition (about 70 %) of CYP2D6-mediated formation of PQ alcohol from (+)-PQ while QN caused only about 45 % inhibition, respectively (Fig. 5).

Other minor metabolites generated by CYP2D6-mediated metabolism of PQ include dihydroxyprimaquine (*m/z* 292) and two that appear to be quinone-imine derivatives of dihydroxyprimaquine (*m/z* 290). All of these minor metabolites were completely suppressed by CQ and QN.

### Influence of CQ and QN on MAO-dependent PQ metabolites

The metabolism of PQ by MAO-A showed a marked enantioselectivity, with (−)-PQ being depleted four times faster than (+)-PQ (Fig. 2b). This depletion was not significantly affected by CQ or QN with any of the substrates until 1 h, after which a very modest inhibition was observed (Fig. 5a–c). The only major metabolite from MAO-dependent metabolism was the alcohol formed by oxidative deamination of the terminal amine. It is formed twice as much from (−)-PQ compared to (+)-PQ. By 2 h, CQ and QN caused about 60 % inhibition of the formation of PQ alcohol with both PQ enantiomers (Fig. 5d–f).

### Influence of CQ and QN on PQ metabolites generated from human hepatocytes

PQ depletion and metabolite production from hepatocyte incubation monitored via UHPLC-MS/MS demonstrated a faster metabolic rate for (−)-PQ as compared to (+)-PQ. The rate of metabolism was profiled against time, yielding an initial half-life of 3.65 and 1.08 h for (+)- and (−)-PQ, and elimination rate constants (ʎ) of 0.19 and 0.64 h^−1^, respectively. The calculated in vitro intrinsic clearance (Cl_int_) was 2.55 and 8.49 (µL/min)/million cells for (+) and (−)-PQ, respectively. Metabolism was observed via oxidative deamination of the terminal amine, hydroxylation of the quinoline ring, and what appears to be a third path, carbamoylation and glucuronidation of the terminal amine (*m/z* 480). The major deaminated metabolites were CPQ, PQ terminal alcohol, a cyclized side chain derivative from the aldehyde (*m/z* 241), cyclized carboxylic acid derivative (*m/z* 257), a quinone-imine product of hydroxylated CPQ (*m/z* 289), CPQ glucuronide (*m/z* 451), and the glucuronide of PQ alcohol (*m/z* 437). All were preferentially generated from the (-)-PQ. The major quinoline oxidation product (*m/z* 274) and 4-hydroxyprimaquine were preferentially formed from (+)-PQ. A prominent conjugate (*m/z* 422) (seemingly a glycosylated PQ, (but perhaps reflecting a fragment or degradation product of another metabolite) was also preferentially (+)-PQ-derived. The PQ carbamoyl glucuronide metabolite (*m/z* 480) accumulated linearly over the incubation period, and was exclusively generated from (+)-PQ.

**Fig. 6:**
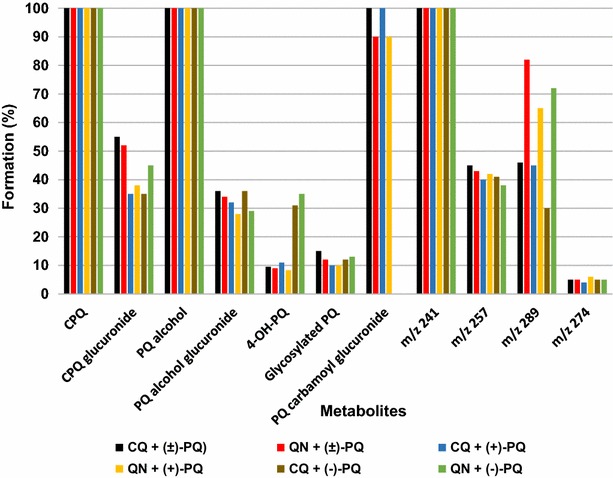
The inhibitory effects of chloroquine and quinine on the formation of metabolites from primaquine and its (+)- and (−)-enantiomers after one-hour incubation in human hepatocytes. Each *point* represents mean of 4 determinations. The major metabolites were carboxyprimaquine [predominantly from (−)-PQ], carbamoyl primaquine glucuronide [exclusively from (+)-PQ] and glycosylated primaquine (formed from both enantiomers). The inhibitory effect of chloroquine and quinine on the formation of carboxyprimaquine and carbamoyl primaquine glucuronide was mild

CQ and QN had no effect on the primary amine oxidation products (CPQ or PQ alcohol or the cyclized aldehyde) (Fig. 6). However, surprisingly they did inhibit substantially the appearance of the glucuronides of CPQ and PQ alcohol. They also inhibited the appearance of the *m/z* 257 metabolite with a similar pattern, suggesting that it may be derived from the CPQ conjugate. The formation of 4-OH-PQ and the quinone-imine (*m/z* 274) were essentially completely suppressed, and the *m/z* 422 metabolite was very strongly suppressed (90–95 %) by both CQ and QN. The apparent quinone-imine of CPQ (*m/z* 289) was partially suppressed, but more prominently by CQ than QN. The carbamoyl glucuronide of PQ (*m/z* 480) was not affected.

## Discussion

The unique therapeutic indications for PQ and the absence of alternatives have made it indispensable in the treatment of *P. vivax* malaria, despite its known haemotoxicity in individuals with genetic deficiency in G6PD. The efficacy and the haemotoxicity of PQ, as well as other 8-AQs, appears to be related to active metabolites, although the specific pathways and metabolites responsible, as well as their sites of formation and mechanisms of action, remain unclear.

Following earlier substantive clinical reports [13, 15, 16] that CQ and QN are capable of altering the therapeutic and toxicological responses to 8-AQs, including PQ, and from recent findings of new human metabolites of PQ [9, 21], it is important to understand how CQ and QN might alter human PQ metabolism. The present study aimed to address these issues.

In an overview, PQ is metabolized via two primary phase I pathways, namely: hydroxylation products derived from CYP-mediated oxidation, especially CYP2D6; and oxidative deamination of the side chain affording the terminal alcohol or carboxylic acid, primarily mediated by MAO-A (though CYP2D6 can also produce the PQ alcohol). In addition, a number of glucuronide conjugates of these phase I metabolites are formed, and an unusual (but not unknown, [26, 27]) carbamoylation/glucuronidation of the parent is observed [21]. CYP2D6-mediated metabolism of PQ is known to produce mono- and dihydroxylated primaquines and their quinone-imine products [9]. In human hepatocytes, a more limited suite of metabolites was observed, with the ring oxidation products quinone-imine *m/z* 274 and the 4-OH-PQ predominant from (+)-PQ, while the oxidation of the terminal amine was the major metabolic route with (−)-PQ [21]. In addition, the PQ-carbamoyl-glucuronide was exclusively formed from (+)-PQ.

Another metabolite (*m/z* 422, apparent PQ-glucoside) seems to be predominantly derived from (+)-PQ, perhaps indirectly. It is of interest in that although it can be formed non-enzymatically, it also appears to arise in hepatocytes by an apparent enzymatic mechanism. Further studies are underway on this metabolite to determine its origin.

The effects of CQ and QN observed in the present studies were most striking for CYP2D6 inhibition, with very modest effects on the oxidative deamination products of MAO. In hepatocytes, the inhibitory effects were most striking on the CYP2D6 related products. The presence of CQ and QN did not appear to significantly inhibit the MAO-dependent pathway in hepatocytes. The inhibitory effects on CYP2D6 appeared to enhance the MAO-mediated metabolism as demonstrated in a mild increase in formation of CPQ, and the metabolites corresponding to *m/z* 241. A surprising inhibition was observed, however, on the formation of the glucuronides of CPQ and PQ alcohol in the human hepatocytes. This would be expected to facilitate the accumulation of the primary oxidative deamination metabolites. This finding is consistent with the report that CQ increased circulating CPQ concentrations when co-administered with PQ in humans [16]. The implications of this are not known, but such metabolites could theoretically exert more pronounced pharmacodynamic effects and may contribute to the observed alteration in PQ response in the presence of CQ and QN.

The profound effects of CQ and QN on the CYP2D6-generated metabolites was not particularly surprising result, since both of these drugs have demonstrated potential for inhibition of CYP2D6 in human studies [18, 28]. The metabolism results from the incubation with human hepatocytes might be presumed to provide a closer picture to in vivo realities. Although CQ/QN suppressed the extent of the formation of a number of metabolites in the human hepatocytes, their inability to alter the clearance and other pharmacokinetic parameters of PQ suggests that the facilitation of one metabolic pathway counters the inhibitory effect on the other. Any observed pharmacodynamic changes therefore could result from the changes in the metabolite profiles.

For moderate CYP2D6 inhibitors like CQ and QN, substrate-specific or orientation/conformation-specific inhibition may be expected. This may explain varying degrees of inhibition on the individual CYP2D6 metabolites of PQ or the individual enantiomer substrates. However, such dramatic inhibition of the formation of the mono-hydroxylated primaquines as was observed at the early time points may have significant pharmacodynamic implications. This might favour alternative metabolic pathways leading to significant changes in therapeutic response, and may play major roles in the earlier observation of mitigated toxicity when PQ was combined with CQ and QN.

However, it seems difficult to harmonize these findings with the well-demonstrated enhancement of PQ anti-malarial efficacy by QN and CQ and the recent observations suggesting that CYP2D6 activity is essential to the anti-malarial efficacy of PQ [17, 29]. Overall, it raises the question of other mechanisms of action for these drugs, and perhaps important roles of other PQ metabolites that are not CYP2D6-dependent. For example, CQ and QN may affect transport processes for cellular uptake or extrusion of PQ or metabolites in a manner that could affect the overall efficacy or toxicity of PQ. Or the pathway to hydroxylated PQ products from other CYPs could be favoured, yielding active metabolites not seen in the absence of CQ or QN. In addition, the abundance of deaminated products in the presence of CQ/QN might be shunted into pathways that yield active metabolites with reduced toxicity. All of these possibilities must await further study, and especially the study in the clinic, where the interaction of PQ with QN and CQ can be assessed in terms of new knowledge of PQ metabolites and new methods for quantitating these.

## Conclusions

This study has been able to assess the influence of CQ/QN on the multiple pathways of PQ metabolism. The identified PQ metabolites showed products of CYP-dependent ring oxidation, MAO-catalyzed oxidation of the terminal amine and phase II conjugation products. Both CQ and QN strongly inhibited the ring oxidation pathway. The suppression of CYP-generated metabolites in favor of the oxidative deamination of the side chain and the abundance of the resultant deaminated metabolites may raise the question of the roles of these metabolites in PQ activity and toxicity. The phase II reactions were also moderately inhibited by CQ/QN. The oxidation of the terminal amines and associated metabolites were generally not influenced by the presence of CQ and QN.

## Abbreviations

8-AQ: 8-aminoquinoline
Cl_int_: intrinsic clearance
CPQ: carboxyprimaquine
CQ: chloroquine
CYP2D6: cytochrome P450 2D6
G6PD: glucose-6-phosphate dehydrogenase
IR: infra-red
MAO: monoamine oxidase
NMR: nuclear magnetic resonance
PQ: primaquine
QN: quinine
